# The Effect of Consuming Carbohydrate With and Without Protein on the Rate of Muscle Glycogen Re-synthesis During Short-Term Post-exercise Recovery: a Systematic Review and Meta-analysis

**DOI:** 10.1186/s40798-020-00297-0

**Published:** 2021-01-28

**Authors:** Jonathan Craven, Ben Desbrow, Surendran Sabapathy, Phillip Bellinger, Danielle McCartney, Christopher Irwin

**Affiliations:** 1 School of Allied Health Sciences, Griffith University, Southport, 4222 Queensland Australia; 2 Queensland Academy of Sport, Nathan, Queensland Australia; 3 Griffith Sports Physiology and Performance, Griffith University, Gold Coast, Queensland Australia; 4 School of Psychology, Faculty of Science, University of Sydney, Sydney, New South Wales Australia

**Keywords:** Nutrition, Glycogen replenishment, Athletes

## Abstract

**Background:**

Rapid restoration of muscle glycogen stores is imperative for athletes undertaking consecutive strenuous exercise sessions with limited recovery time (e.g. ≤ 8 h). Strategies to optimise muscle glycogen re-synthesis in this situation are essential. This two-part systematic review and meta-analysis investigated the effect of consuming carbohydrate (CHO) with and without protein (PRO) on the rate of muscle glycogen re-synthesis during short-term post-exercise recovery (≤ 8 h).

**Methods:**

Studies were identified via the online databases Web of Science and Scopus. Investigations that measured muscle glycogen via needle biopsy during recovery (with the first measurement taken ≤ 30 min post-exercise and at least one additional measure taken ≤ 8 h post-exercise) following a standardised exercise bout (any type) under the following control vs. intervention conditions were included in the meta-analysis: part 1, water (or non-nutrient beverage) vs. CHO, and part 2, CHO vs. CHO+PRO. Publications were examined for methodological quality using the Rosendal scale. Random-effects meta-analyses and meta-regression analyses were conducted to evaluate intervention efficacy.

**Results:**

Overall, 29 trials (*n* = 246 participants) derived from 21 publications were included in this review. The quality assessment yielded a Rosendal score of 61 ± 8% (mean ± standard deviation). Part 1: 10 trials (*n* = 86) were reviewed. Ingesting CHO during recovery (1.02 ± 0.4 g·kg body mass (BM)^−1^ h^−1^) improved the rate of muscle glycogen re-synthesis compared with water; change in muscle glycogen (MG_Δ_) re-synthesis rate = 23.5 mmol·kg dm^−1^ h^−1^, 95% CI 19.0–27.9, *p* < 0.001; *I*^2^ = 66.8%. A significant positive correlation (*R*^*2*^ = 0.44, *p* = 0.027) was observed between interval of CHO administration (≤ hourly vs. > hourly) and the mean difference in rate of re-synthesis between treatments. Part 2: 19 trials (*n* = 160) were reviewed. Ingesting CHO+PRO (CHO: 0.86 ± 0.2 g·kg BM^−1^ h^−1^; PRO: 0.27 ± 0.1 g·kg BM^−1^ h^−1^) did not improve the rate of muscle glycogen re-synthesis compared to CHO alone (0.95 ± 0.3 g·kg BM^−1^ h^−1^); MG_Δ_ re-synthesis rate = 0.4 mmol·kg  dm^−1^ h^−1^, 95% CI −2.7 to 3.4, *p* = 0.805; *I*^2^ = 56.4%.

**Conclusions:**

Athletes with limited time for recovery between consecutive exercise sessions should prioritise regular intake of CHO, while co-ingesting PRO with CHO appears unlikely to enhance (or impede) the rate of muscle glycogen re-synthesis.

**Trial Registration:**

Registered at the International Prospective Register of Systematic Reviews (PROSPERO) (identification code CRD42020156841).

**Supplementary Information:**

The online version contains supplementary material available at 10.1186/s40798-020-00297-0.

## Key Points

- Carbohydrate provision enhances muscle glycogen re-synthesis compared to no nutritional provision.
- Co-ingestion of protein with carbohydrate does not enhance muscle glycogen re-synthesis compared to consuming carbohydrate alone.
- The interval of carbohydrate administration was found to be an influential factor on the rate of muscle glycogen re-synthesis.

## Introduction

Restoration of both muscle and liver glycogen stores is a critical element of post-exercise recovery [1, 2]. It is particularly important for athletes training multiple times per day, since sessions are often separated by short periods of recovery (i.e. ≤ 8 h) and inadequate muscle and liver glycogen restoration can impair subsequent athletic performance [2, 3]. As such, a considerable amount of research has been directed towards identifying nutritional strategies that facilitate optimal post-exercise glycogen repletion [4–7].

Carbohydrate (CHO) is the main substrate for muscle glycogen re-synthesis [1]. Thus, the impact of various CHO manipulations on restoration of muscle glycogen stores has been a research priority, including the amount of CHO provided [8]; type and combination of mono-saccharides administered [9, 10]; molecular weight of CHO provided [11]; timing of CHO intake relative to the completion of exercise [12, 13]; form of delivery (e.g. liquid vs. solid) [14, 15]; and provision of other nutrients [16–25], most notably protein (PRO) [26–29]. This work has been summarised in a number of narrative reviews [4–7, 30] and has also led to the development of sports nutrition guidelines describing best-practice approaches for athlete recovery [31, 32]. These guidelines suggest that following exercise, athletes should consume between 1.0 and 1.2 g CHO·kg of body mass (BM)^−1^ h^−1^ to optimise the rate of muscle glycogen re-synthesis [32]. The addition of PRO (0.3–0.4 g there should be a dot here 'g·kg' line 79 kg BM^−1^ h^−1^) has also been proposed to increase the rate of muscle glycogen re-synthesis when CHO consumption is sub-optimal (≤ 0.8 g·kg BM^−1^ h^−1^) [30]. However, these guidelines have been formulated based on original research and narrative reviews. Synthesising the available literature using meta-analytical techniques will quantify overall effects and assess whether these current guidelines are supported.

Of course, it is important to consider the influence of other contextual factors (other than CHO manipulation and the addition of PRO) on the rate of muscle glycogen re-synthesis during short-term post-exercise recovery. Some noteworthy contextual factors include training status [33], types of muscle contraction during exercise [34], muscle fibre typology [35–38], and the degree of muscle glycogen depletion induced by the initial exercise bout [39, 40]. However, the extent to which these factors moderate the effect of CHO provision on the rate of muscle glycogen re-synthesis during short-term post-exercise recovery has yet to be clarified meta-analytically.

The aim of the present review was to examine the effects of consuming: (1) CHO (in isolation) and (2) CHO+PRO on the rate of muscle glycogen re-synthesis during short-term exercise recovery using meta-analytic techniques. The extent to which other contextual factors (e.g. CHO- dose, timing) influence the rate of muscle glycogen re-synthesis are explored.

## Methods

The methodology of this review was developed in accordance with the *Preferred Reporting Items for Systematic Reviews and Meta-Analysis Protocols 2015 Statement* [41] and registered at the International Prospective Register of Systematic Reviews (PROSPERO) (identification code: CRD42020156841) before beginning the formal study selection process.

### Literature Search

**Fig. 1:**
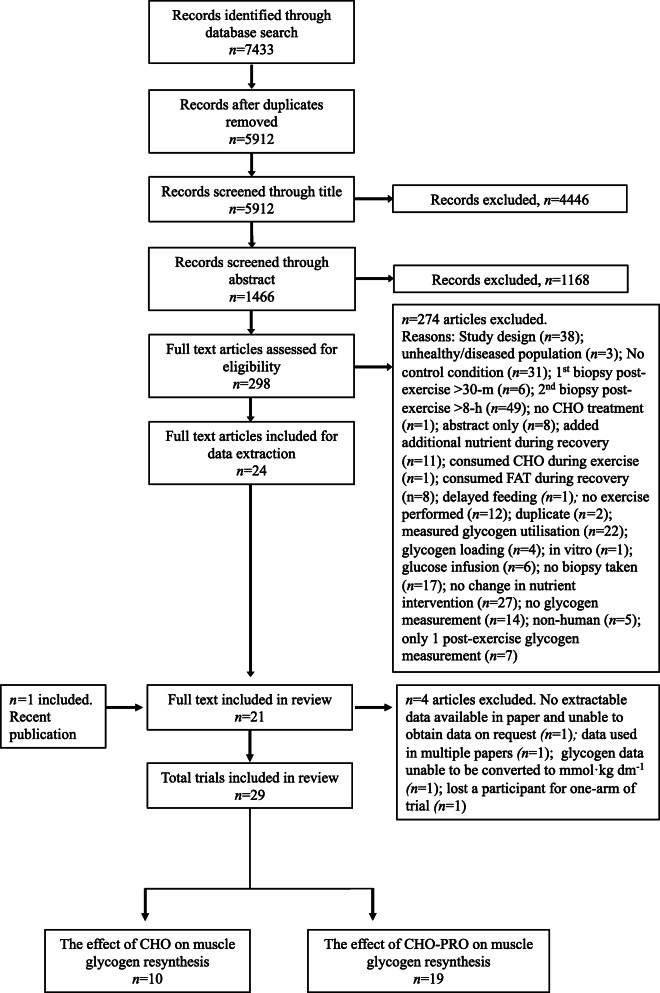
PRISMA flow chart (study selection methodology). Where a study contained more than one intervention-arm, the separate arms were treated as discrete ‘studies’, termed as ‘trials’

Original research studies were identified by searching the online databases ‘Web of Science (via Thomas Reuters)’ and ‘Scopus’ from inception until September 2019 using the Boolean expression: (exercis* AND glycogen) OR (postexercis* AND glycogen). The star symbol (*) was used to capture derivatives (by suffixation) of the search terms (e.g. exercised). No other search restrictions were imposed. A final search was also conducted in March 2020 to capture any recent publications. One manuscript [42] was identified in this search for inclusion. Two investigators (JC and CI) independently screened potential studies to identify relevant texts. Initially, all irrelevant titles were discarded. The remaining articles were then systematically screened for eligibility by abstract and full text. The decision to include or discard potential research studies was made between two investigators (JC and CI). Any discrepancies were resolved in consultation with a third investigator (BD). The reference lists of all included studies were hand-searched for missing publications. Full details of the screening process are illustrated in Fig. 1.

### Inclusion and Exclusion Criteria

**Fig. 2:**
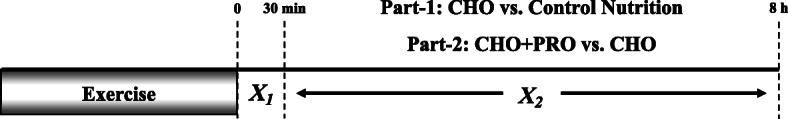
Schematic of the experimental protocol used in studies that were eligible for inclusion in the current review. Crosses (*X*_1_ and *X*_2_) represent the pre-treatment and post-treatment needle biopsies, respectively

Original research studies that met the following criteria were included in this review:

1. Controlled trials employing repeated-measures experimental designs.
2. Human studies on adult (≥ 18 years of age) men and women with no known medical conditions and co-morbidities.
3. Muscle glycogen concentrations were measured by needle biopsy (the ‘gold standard’ measurement technique) [43] under both control and intervention conditions (refer to the ‘Control and intervention conditions’ section), with the first (i.e. ‘pre-treatment’) measurement taken ≤ 30 min post-exercise and at least one additional (i.e. ‘post-treatment’) measurement taken ≤ 8 h post-exercise; a schematic of the experimental protocol employed in eligible studies is illustrated in Fig. 2.
4. Full-text original research studies were published in English; all other documents were discarded.

Studies were excluded from the review if:

1. A between-subject experimental design was employed.
2. Energy containing dietary constituents other than CHO and PRO (e.g. alcohol, fat) or ergogenic substances (e.g. caffeine, creatine) were administered post-exercise.
3. Exercise was not standardised across trials (or no exercise was performed).
4. Dietary constituents other than water were administered during exercise.
5. Treatments were not administered orally.
6. Muscle glycogen concentrations were not measured by needle biopsy (e.g. nuclear magnetic resonance spectroscopy, ultrasound).
7. Muscle glycogen data were not adequately reported (i.e. mean ± standard deviation (SD) was not reported and could not be calculated).

In the event that data were not adequately reported, the corresponding author was contacted via email in an attempt to retrieve the missing data. Where data were presented in graphical format only, a web-based tool (‘WebPlotDigitizer’, https://apps.automeris.io/wpd/) was used to extract numeric values.

Several publications identified via the literature search contained more than one intervention and control comparison that was eligible for inclusion. In these instances, the separate study arms were treated as individual investigations, and termed ‘trials’. Separate trials derived from a single research study are denoted by the addition of a lower-case letter (i.e. a–c) to the citation.

### Control and Intervention Conditions

The present systematic review and meta-analysis compared the intervention and control conditions via a two-part investigation: (1) CHO vs. control (i.e. water or a non-nutritive placebo treatment) and (2) PRO (including isolated or mixed amino-acids (AA), e.g. glutamine, leucine) co-ingested with CHO (CHO+PRO) vs. CHO.

### Primary and Secondary Research Outcomes

The primary research outcome in this investigation was the rate of muscle glycogen re-synthesis. Values were initially extracted in mmol·kg of dry mass (dm)^−1^, or in mmol·kg of wet mass^−1^, and then multiplied by a factor of 4.35 to convert to mmol·kg dm^−1^, as described by Areta and Hopkins [44]. Where the rate of muscle glycogen re-synthesis was not reported directly (or could not be calculated using raw data supplied by authors) [26, 28, 45–47], but pre- and post-treatment muscle glycogen concentrations were known, the following methods were used to determine the missing values. First, the change in muscle glycogen concentration (MG_∆_) (i.e. total amount of glycogen re-synthesised) was calculated for the control and intervention conditions. The SD of this (within-trial) change (SD_∆_) was then calculated using the following formula [48]:

$$ {\mathrm{SD}}_{\Delta}=\sqrt{\left({{\mathrm{SD}}^2}_{\mathrm{post}-\mathrm{exercise}\ \mathrm{MG}}+{{\mathrm{SD}}^2}_{\mathrm{end}\ \mathrm{of}\ \mathrm{recovery}\ \mathrm{MG}}\ \right)-\left(2\ \mathrm{x}\ R\ \mathrm{x}\ {\mathrm{SD}}_{\mathrm{post}-\mathrm{exercise}\ \mathrm{MG}}\times {\mathrm{SD}}_{\mathrm{end}\ \mathrm{of}\ \mathrm{recovery}\ \mathrm{MG}}\right)} $$

where *R* is the mean correlation coefficient calculated using raw data derived from four CHO vs. control trials (CHO: *R* = 0.62; control: *R* = 0.64) [42, 49–51] and 17 CHO+PRO vs. CHO trials (CHO+PRO: *R* = 0.79; CHO: *R* = 0.76) [27, 29, 52–60]. The rate of muscle glycogen re-synthesis under each condition was then determined by dividing the total amount of glycogen re-synthesised (i.e. the mean and SD values) by the length of the recovery period (i.e. time between biopsies).

### Quality Assessment

Included studies were examined for methodological quality using the Rosendal Scale [61], where excellent methodological quality is indicated by a Rosendal score ≥ 60% [62]. Scoring was determined by dividing the number of ‘yes’ responses by the total number of applicable items. Studies with a Rosendal score < 50% are typically excluded from reviews owing to their increased risk of experimental bias; however, no study received a score < 50% in the current analysis.

### Data Extraction

Data were extracted in accordance with the Cochrane Handbook for Systematic Reviews of Interventions *Checklist of Items to Consider in Data Collection or Data Extraction* [48] and entered into a Microsoft Excel spreadsheet. Extracted data included (1) participant characteristics (e.g. athletic population, age, BM, sex, aerobic power ($\dot{V}$O_2peak_)); (2) pre-trial standardisation procedures; (3) exercise mode and protocol used to reduce muscle glycogen concentrations; (4) the treatment administration protocol (i.e. the delivery medium, amount, type, timing); (5) timing and number of muscle biopsies; and (6) muscle glycogen concentrations and, where provided, rate of re-synthesis (mmol·kg dm^−1^ h^−1^).

### Statistical Analyses

All statistical procedures were performed using SPSS, Version 26.0 (Armonk, NY: IBM Corp) and Comprehensive Meta-Analysis, Version 3.0 (Englewood, NJ: Biostat Inc). Weighted mean effect estimates and meta-regression coefficients are presented as mean (95% confidence interval (95% CI) or range). All other data are presented as mean ± SD unless stated otherwise.

#### Weighted Mean Effect

Meta-analyses were performed to determine the influence of (1) CHO vs. control (water or a non-nutritive placebo treatment) and (2) CHO+PRO vs. CHO on the rate of muscle glycogen re-synthesis. Individual effect sizes were calculated as the raw mean difference (i.e. mmol·kg dm^−1^ h^−1^), where positive effect estimates reflect higher rates of muscle glycogen re-synthesis with the intervention condition. Where the SD of this between-trial change was not reported directly (or was unable to be calculated using raw data supplied by the authors) [8, 26–29, 42, 45–47, 49–51, 57–60], the missing value was imputed using the following formula [48]:

$$ {\mathrm{SD}}_{\Delta}=\sqrt{\left({{\mathrm{SD}}^2}_{\mathrm{Control}}+{{\mathrm{SD}}^2}_{\mathrm{Intervention}}\ \right)-\left(2\ \mathrm{x}\ R\ \mathrm{x}\ {\mathrm{SD}}_{\mathrm{Control}}\times {\mathrm{SD}}_{\mathrm{Intervention}}\right)} $$

In this case, *R* was approximated as 0.28 using raw data from eight CHO+PRO vs. CHO trials [52–56]; the same *R* value of 0.28 was used for the CHO vs. control comparison (as no raw data from these trials could be obtained to determine an independent value). Sensitivity analyses were performed using *R* = 0.60 and 0.90 to test the robustness of the imputed value. In addition, trials were individually excluded to examine the influence of their removal on the overall effect estimate.

Weighted mean treatment effects were calculated using random-effect models, where trials were weighted by the inverse variance for the change in the outcome measure (i.e. rate of muscle glycogen re-synthesis). Statistical significance was attained if the 95% CI did not include zero. Heterogeneity was assessed using Cochran’s *Q* and the *I*^2^ index. Low, moderate, and high heterogeneity was indicated by an *I*^2^ value of 25, 50, and 75%, respectively [63]. A *p* value < 0.10 for Cochran’s *Q* was used to indicate significant heterogeneity [48].

#### Meta-regression Analysis

Restricted maximum likelihood, random-effects simple meta-regression analyses were performed to determine whether the magnitude of difference in the rate of muscle glycogen re-synthesis between treatments was influenced by: (1) dose of CHO provided (relative and absolute); (2) pre-treatment muscle glycogen concentrations (i.e. ≤ 150 mmol·kg dm^−1^ vs. > 150 mmol·kg dm^−1^, as it has been hypothesised that levels ≤ 150 mmol·kg dm^−1^ can potentially accelerate muscle glycogen re-synthesis [4, 7]); (3) the relative difference in pre-treatment muscle glycogen concentrations (%); (4) interval of CHO administration during recovery (i.e. ≤ hourly vs. > hourly); (5) amount of CHO provided (i.e. met recommended guidelines (≥ 1.0 g·kg BM^−1^·h^−1^) or not); (6) mode of exercise; (7) difference in the energy content of the CHO+PRO and CHO treatments (i.e. magnitude of energy difference between treatments); (8) PRO source (i.e. whole PRO vs. single (or mixture) AA; (9) providing PRO at a rate ≥ 0.3 g·kg BM^−1^·h^−1^ when the amount of CHO was sub-optimal (≤ 0.8 g·kg BM^−1^ h^−1^) but matched in control (i.e. CHO only) treatment; and (10) methodological quality (Rosendal score %) of the study. At least 10 data points were required for a variable to qualify for meta-regression analysis. Regression analyses were examined for influential cases and outliers (i.e. studentised residuals, Cook’s distance and centred leverage values) and multicollinearity (variance inflation factor). Statistical significance was accepted as *p* < 0.05.

## Results

### Overview of Included Studies and Study Quality

The literature search initially identified 25 eligible investigations. However, four of these had to be excluded because the muscle glycogen data (1) could not be extracted (or retrieved) [13]; (2) were the same as those reported in an earlier publication [64] that was already included [50]; (3) incorporated the results of one participant that did not complete both treatments (i.e. was not a within-subject comparison) [65]; and (4) were reported in a metric that could not be reliably converted to mmol·kg dm^−1^ [66] using the factors outlined by Areta and Hopkins [44]. Overall, 29 trials (*n* = 246 participants) derived from 21 publications were included in this review. Methodological quality assessment yielded an average Rosendal score of 61 ± 8%; all trials scored ≥ 50% (range 50–77%). Results of the quality assessment are shown in Supplementary Table S1.

### Effect of CHO vs. Control (Water or Other Non-nutritive Treatment) on Muscle Glycogen Re-synthesis Rate

Ten trials (*n* = 86; 91% men) derived from nine publications investigated the effect of CHO on the rate of muscle glycogen re-synthesis during post-exercise recovery. Eight [8, 26, 28, 42, 45, 46, 50] used cycling and two [49, 51] used resistance training as the mode of glycogen-depleting exercise. The mean relative CHO intake was 1.02 g·kg BM^−1^ h^−1^ (range 0.50–1.5 g·kg BM^−1^ h^−1^), and the mean post-exercise recovery time was 2.9 h (range 1.0–5.0 h). On average, participants’ post-exercise (i.e. pre-treatment) muscle glycogen concentrations were 179 mmol·kg dm^−1^ (range 12–406 mmol·kg dm^−1^) and 197 mmol·kg dm^−1^ (range 30–441 mmol·kg dm^−1^) for the CHO and control conditions, respectively. Characteristics of the included trials are summarised in Table 1.

**Table 1:** Characteristics of included trials evaluating post-exercise muscle glycogen re-synthesis during short-term recovery (≤ 8 h) with provision of CHO vs. control

Study	Participants	Mean $\dot{\boldsymbol{V}}{\mathbf{O}}_{\mathbf{2peak}}$ **(mL kg min**^**−1**^**)**	Fasted vs. Fed	Exercise protocol	End of exercise glycogen – control (mmol·kg dm^**−1**^)	End of exercise glycogen – intervention (mmol·kg dm^**−1**^)	Source of CHO	Timing of treatment (h)	CHO (g·kg BM^**−1**^ h^**−1**^)	Recovery time (h)	Mean difference in re-synthesis rate (mmol·kg dm^**−1**^ h^**−1**^)
Mathai et al. [46]	7 (7 M)	48.4	Fed	Cycled to exh at 65% $\dot{V}$O_2peak_	146	82	Maltodextrin + dextrose	0, 1	1.0	2	59.0
Pascoe et al. [49]	8 (8 M)	N.S	Fasted	Resistance training: 6 single leg knee ext at 70% 1RM/set x ~ 9 set with 30 s recovery between set	441	399	Glucose polymers + sucrose	0, 1	1.5	2	47.8
Cheng et al. [42]	8 (8 M)	48.5	Fasted	Cycled for 1 h at 60% $\dot{V}$O_2peak_; followed by 4 × 30 s all-out sprints	30	12	Glucose + fructose	0, then every 0.25 (until 2.5)	1.5	3	36.1
Tarnopolsky et al. [26]	16 (8 M)	M: 56.9 F: 51.7	Fasted	Cycled for 1.5 h at 65% $\dot{V}$O_2peak_	210	163	Sucrose + glucose polymer	0, 1	0.5	4	30.6
van Hall et al. [28]a	5 (N.S)	61.0	N.S	Cycled to exh: 2-min intervals alternating between 50 and 90% $\dot{V}$O_2peak_	78	90	Sucrose	0, then every 0.25 (until 3.75)^a^	1.2	4	28.8
Wilson et al. [50]	10 (10 M)	54.8	Fed	Cycled to exh at 65% of $\dot{V}$O_2peak_	74	109	Dextrose	0, 1, 2, 3, 4	1.0	5	25.1
Ivy et al. [8]b	8 (8 M)	52.2	Fasted	Cycled for 2 h: 15-min intervals alternating between 62 and 75% $\dot{V}$O_2peak_	158	139	Glucose polymer	0	1.5	2	19.1
Roy and Tarnopolsky [51]	10 (10 M)	N.S	Fed	Resistance training: whole-body workout; ~ 10 rep at ~ 80% 1RM /set x 3 set x 9 exercises + 20 sit-ups	248	235	Sucrose + glucose polymer	0, 1	0.5	4	17.3
Ivy et al. [8]a	8 (8 M)	52.2	Fasted	Cycled for 2 h: 15-min intervals alternating between 62 and 75% $\dot{V}$O_2peak_	158	156	Glucose polymer	0	0.75	2	16.5
Haub et al. [45]	6 (6 M)	50.8	Fed	Cycled: 100 kJ test (at 90% peak resistance achieved during GXT)	429	406	Maltodextrin	0	0.7	1	14.4

All trials tabulated used a randomised, controlled, within-subject experimental design. Fasted vs. Fed: whether participants were fed or fasted prior to exercise. ‘a’ and ‘b’ refer to separate trials derived from a single research study

*N.S* not specified, *M* male, *F* female, *exh* exhaustion, *Rep* repetition, *ext* extension, *RM* repetition max, *min* minute, $\dot{V}$*O*_*2peak*_ peak maximal oxygen uptake, *BM* body mass, *dm* dry mass, *kJ* kilojoule, *GXT* graded exercise test

^a^Estimated last beverage was consumed at 3.75 h

**Fig. 3:**
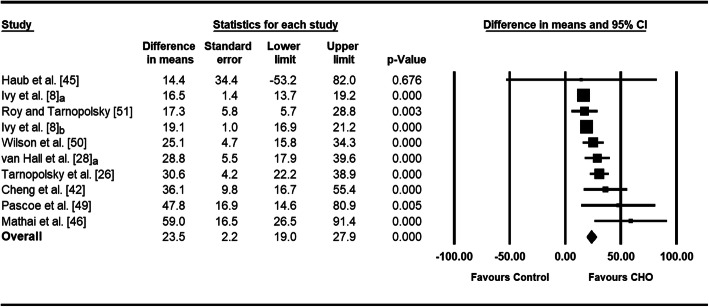
Forest plot displaying the effect of CHO vs. control (non-nutrient treatment) on rate of muscle glycogen re-synthesis during short-term recovery. The size of the squares is proportional to the weight of the study. A positive effect estimate indicates greater rate of muscle glycogen replenishment with CHO than control. Note: ‘a’ and ‘b’ refer to separate trials derived from a single research study

The overall weighted mean effect estimate indicated that CHO administration significantly increased the rate of muscle glycogen re-synthesis during short-term post-exercise recovery (MG_Δ_ re-synthesis rate = 23.5 mmol·kg dm^−1^ h^−1^, 95% CI 19.0–27.9, *p* < 0.001; *I*^2^ = 66.8%) (Fig. 3). The magnitude and statistical significance of the effect were stable during sensitivity analyses where trials were removed (MG_Δ_ re-synthesis rate ranged from 21.8 to 26.8 mmol·kg dm^−1^ h^−1^ and 95% CIs did not include zero). Findings were also comparable when alternative correlation coefficients were used (Supplementary Table S2).

Simple meta-regression analyses identified a significant, positive association between the mean difference in muscle glycogen re-synthesis rate and the interval of CHO administration, such that studies providing CHO more frequently (i.e. ≤ hourly) induced a higher rate of muscle glycogen re-synthesis than those providing CHO less frequently (i.e. > hourly) (*R*^*2*^ = 0.44, *p* = 0.027). No significant associations were identified between the mean difference in muscle glycogen re-synthesis rate and any other contextual factors (Table 2).

**Table 2:** The influence of contextual factors on the mean difference in rate of muscle glycogen re-synthesis (analysed via restricted maximum likelihood, simple meta-regression) for CHO vs. control treatments

Effect estimate	Mean difference (re-synthesis rate mmol·kg dm^−1^ h^−1^)	
Covariate	Coefficient (95% CI)	*p* value
Glycogen post-exercise (≤ 150 mmol·kg dm^−1^ vs. > 150 mmol·kg dm^−1^)	4.32 (− 7.78 to 16.4)	0.484
Interval of CHO administration ≤ hourly (yes vs. no)	11.0 (1.24 to 20.9)	0.027
Total CHO (g) intake	− 0.00 (− 0.06 to 0.04)	0.665
Relative CHO (g·kg BM^−1^ h^−1^) intake	5.00 (− 11.5 to 20.5)	0.553
Met CHO guidelines (yes vs. no)	7.05 (− 5.26 to 19.4)	0.262
Length of recovery (h)	2.09 (− 1.98 to 6.17)	0.314
Relative difference in muscle glycogen immediately post-exercise	− 0.12 (− 0.35 to 0.11)	0.317
Mode of exercise (cycling vs. resistance)	1.84 (− 15.3 to 19.0)	0.833
Study quality (Rosendal score %)	0.92 (− 0.10 to 1.94)	0.076

*CHO* carbohydrate, *BM* body mass, *dm* dry mass

### Effect of CHO+PRO vs. CHO on Muscle Glycogen Re-synthesis Rate

Nineteen trials (*n* = 160; 96% men) derived from 13 publications investigated the effect of co-ingesting CHO with PRO on the rate of muscle glycogen re-synthesis during post-exercise recovery. Seventeen [27–29, 47, 54–60] used cycling and two [52, 53] used running as the mode of glycogen-depleting exercise. Mean post-exercise muscle glycogen concentrations were 131 mmol·kg dm^−1^ (range 85–233 mmol·kg dm^-1^) and 129 mmol·kg dm^−1^ (range 64–235 mmol·kg dm^−1^) for the CHO and CHO+PRO trials, respectively. The mean relative intake of CHO was 0.95 g·kg BM^−1^ h^−1^ (range 0.60–1.6 g·kg BM^−1^ h^−1^) for the CHO trials and 0.86 g·kg BM^−1^ h^−1^ (range 0.50–1.2 g·kg BM^−1^ h^−1^) for the CHO+PRO trials. The mean relative PRO intake was 0.27 g·kg BM^−1^ h^−1^ (range 0.05–0.40 g·kg BM^−1^ h^−1^) for the CHO+PRO trials and the mean energy difference (kJ) between trials (favouring CHO+PRO) was 884 kJ (range 0–2343 kJ). Characteristics of the included trials are summarised in Table 3.

**Table 3:** Characteristics of included trials evaluating post-exercise muscle glycogen re-synthesis during short-term recovery (≤ 8 h) with provision of CHO + PRO vs. CHO

Study	Participants	Mean $\dot{\boldsymbol{V}}{\mathbf{O}}_{\mathbf{2peak}}$ **(mL·kg·min**^**−1**^**)**	Exercise protocol	Post-exercise glycogen (mmol·kg dm^**−1**^) control	Post-exercise glycogen (mmol·kg dm^**−1**^) intervention	Type of CHO+PRO	Timing of treatment (h)	CHO (g·kg BM^**−1**^ h^**−1**^) control	CHO (g·kg BM^**−1**^ h^**−1**^) intervention	PRO (g·kg BM^**−1**^ h^**−1**^) intervention	Recovery time (h)	Mean difference in re-synthesis rate (mmol·kg dm^**−1**^ h^**−1**^)
van Loon et al. [29]a	8 (8M)	N.S	Cycled to exh: 2-min intervals alternating between 50 and 90% $\dot{V}$O_2peak_	190	174	Glucose + maltodextrin + wheat protein hydrolysate + leucine + phenylalanine	0, 0.5, 1, 1.5, 2, 2.5, 3, 3.5, 4, 4.5	0.8	0.8	0.4	5	18.7
Zawadzki et al. [58]	9 (9M)	66.6	Cycled for 2 h alternating between 65 and 85% $\dot{V}$O_2peak_	233	217	Dextrose + maltodextrin + milk + WPI	0, 2	0.76	0.76	0.27	4	10.2
Yaspelkis and Ivy [57]	12 (12M)	67.2	Cycled for 2 h alternating between 60 and 80% $\dot{V}$O_2peak_	144	134	Maltodextrin + arginine	0, 1, 2, 3	1.0	1.0	0.08	4	9.5
van Hall et al. [27]a	8 (8M)	N.S	Cycled to exh: 2 min intervals alternating between 50 and 90% $\dot{V}$O_2peak_	107	74	Glucose + wheat protein hydrolysate	0.25, 1, 2	0.8	0.8	0.3	3	5.8
van Hall et al. [27]b	8 (8M)	N.S	Cycled to exh: 2-min intervals alternating between 50 and 90% $\dot{V}$O_2peak_	107	108	Glucose + WPH	0.25, 1, 2	0.8	0.8	0.3	3	4.9
Alghannam et al. [52]	6 (5M)	64.0	Ran to exh at 70% $\dot{V}$O_2peak_	103	100	Sucrose + WPH	0, 1, 2, 3	1.2	0.8	0.4	4	4.3
Beelen et al. [59]	14 (14M)	61.5	Cycled to exh: 2-min intervals alternating between 50 and 90% $\dot{V}$O_2peak_	172	184	Maltodextrin + glucose + casein hydrolysate + leucine	Every 0.5	1.2	1.2	0.3	6	2.1
Howarth et al. [55]a	6 (6M)	49.3	Cycled for 2 h: 10-min intervals alternating between 50 and 80% $\dot{V}$O_2peak_	97	64	Maltodextrin + WPC	0, then at 0.25 until 3	1.2	1.2	0.4	4	1.9
Betts et al. [53]	6 (6M)	61.0	Ran for 1.5 h at 70% $\dot{V}$O_2peak_	203	235	Sucrose + WPI	0, .5, 1. 1.5, 2, 2.5, 3, 3.5	0.8	0.8	0.3	4	−0.2
Howarth et al. [55]b	6 (6M)	49.3	Cycled for 2 h: 10-min intervals alternating between 50 and 80% $\dot{V}$O_2peak_	85	64	Maltodextrin + WPC	0, then at 0.25 until 3	1.6	1.2	0.4	4	−0.4
Carrithers et al. [47]	8 (8M)	55.7	Cycled for 1.25 h at 70% $\dot{V}$O_2peak_; followed by 6 × 1 min sprint at 125% $\dot{V}$O_2peak_	108	89	Sucrose + fructose + dextrose + mixed AA	0, 0.5, 1, 1.5, 2, 2.5, 3, 3.5	1.0	0.86	0.14	4	−1.3
van Hall et al. [27]c	8 (8M)	N.S	Cycled to exh: 2-min intervals alternating between 50 and 90% $\dot{V}$O_2peak_	107	80	Glucose + glutamine	0.25, 1, 2	0.8	0.8	0.3	3	−2.0
Cogan et al. [54]a	11 (11M)	61.2	Cycled for 2 h at 70% $\dot{V}$O_2peak_	136	134	Glucose + maltodextrin + sodium caseinate	0, 2	0.6	0.5	0.08	4	−2.7
Wang et al. [56]b	10 (7M)	50.2	Cycled for 2 h at 70%$\dot{V}$O_2peak_; followed by 5 × 1 min sprint at 85%$\dot{V}$O_2peak_	114	146	Dextrose + mixed AA	0, 2	0.6	0.6	0.045	4	−3.7
van Hall et al. [28]b	5 (N.S)	61.0	Cycled to exh: 2 min intervals alternating between 50 and 90% $\dot{V}$O_2peak_	90	69	Sucrose + WPH	0, and then every 0.25 (until 3.75)^a^	1.2	1.2	0.36	4	−4.5
Wang et al. [56]a	10 (7M)	50.2	Cycled for 2 h at 70%$\dot{V}$O_2peak_; followed by 5 × 1 min sprint at 85% $\dot{V}$O_2peak_	114	124	Dextrose + mixed AA	0, 2	0.6	0.6	0.09	4	−6.6
Cogan et al. [54]b	11 (11M)	61.2	Cycled for 2 h at 70% $\dot{V}$O_2peak_	136	107	Glucose + maltodextrin + sodium caseinate hydrolysate	0, 2	0.6	0.5	0.08	4	−7.1
van Loon et al. [29]b	8 (8M)	N.S	Cycled to exh: 2-min intervals alternating between 50 and 90% $\dot{V}$O_2peak_	138	174	Glucose + maltodextrin + wheat protein hydrolysate + leucine + phenylalanine	0, 0.5, 1, 1.5, 2, 2.5, 3, 3.5, 4, 4.5	1.2	0.8	0.4	5	−9.6
Jentjens et al. [60]	8 (8M)	63.3	Cycled to exh: 2-min intervals alternating between 50 and 90% $\dot{V}$O_2peak_	106	176	Glucose + maltodextrin + wheat protein hydrolysate + leucine + phenylalanine	0, 0.5, 1, 1.5, 2, 2.5	1.2	1.2	0.4	3	−14.4

All trials tabulated used a randomised, controlled, within-subject experimental design. ‘a’, ‘b’, and ‘c’ refer to separate trials derived from a single research study

*N.S* not specified, *M* male, *min* minute, *exh* exhaustion, $\dot{V}$O_2peak_ peak maximal oxygen uptake, *BM* body mass, *dm* dry mass, *CHO* carbohydrate, *PRO* protein, *AA* amino acids, *WPI* whey protein isolate, *WPC* whey protein concentrate; WPH: whey protein hydrolysate, *mix* mixture

^a^Estimated last beverage was consumed at 3.75 h

**Fig. 4:**
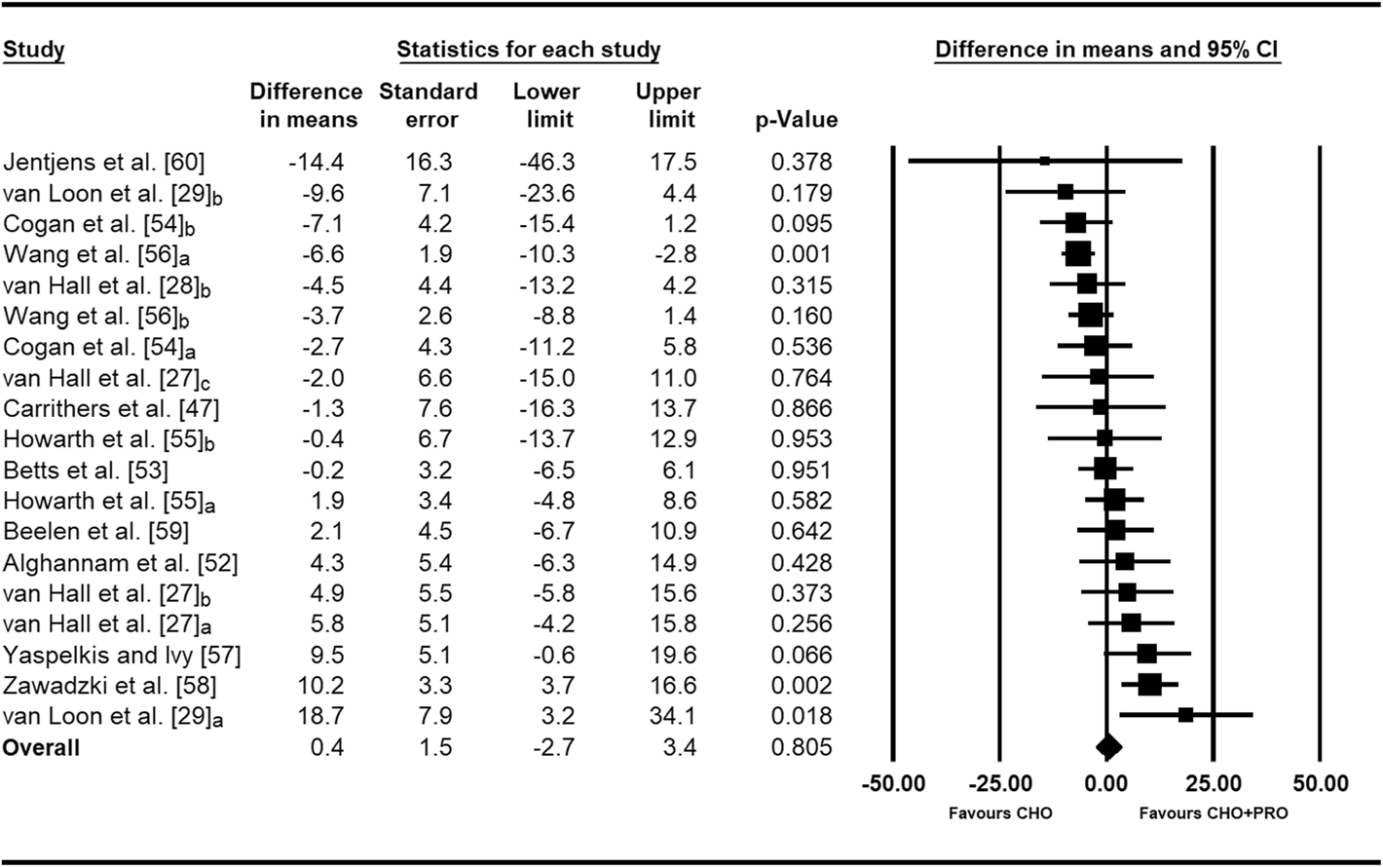
Forest plot displaying the effect of CHO+PRO vs. CHO on rate of muscle glycogen re-synthesis during short-term recovery. The size of the squares is proportional to the weight of the study. A positive effect estimate indicates greater rate of muscle glycogen replenishment with CHO+PRO than CHO. Note: ‘a’, ‘b’, and ‘c’ refer to separate trials derived from a single research study

The weighted mean effect estimate indicated that co-ingesting PRO with CHO did not significantly improve muscle glycogen re-synthesis rate (MG_Δ_ re-synthesis rate = 0.4 mmol·kg dm^−1^ h^−1^, 95% CI −2.7 to 3.4, *p* = 0.805; *I*^2^ = 56.4%) compared to consuming CHO alone (Fig. 4). The magnitude and statistical significance of the effect were stable during sensitivity analyses where trials were individually removed (MG_Δ_ re-synthesis rate (mmol·kg dm^−1^ h^−1^) range −0.6 to 1.0, 95% CIs did include zero). Findings were also comparable when alternative correlation coefficients were used (Supplementary Table S3).

There were no significant relationships identified between the mean difference in rate of muscle glycogen re-synthesis and any of the contextual factors explored using meta-regression. Results of the meta-regression analyses are summarised in Table 4.

**Table 4:** The influence of contextual factors on the mean difference in rate of muscle glycogen re-synthesis (analysed via restricted maximum likelihood, simple meta-regression) for CHO+PRO vs. CHO treatments

Effect estimate	Mean difference (re-synthesis rate mmol·kg dm^−1^ h^−1^)	
Covariate	Coefficient (95% CI)	*p* value
Relative difference in muscle glycogen immediately post-exercise	−0.10 (−0.25 to −0.06)	0.216
Magnitude of energy (kJ) difference between treatments	0.01 (−0.00 to 0.01)	0.084
Whole PRO vs. single/mixed AA	3.46 (−2.84 to 9.77)	0.282
Recovery duration (h)	0.28 (−4.39 to 4.95)	0.906
Mode of exercise (running vs. cycling)	1.33 (−8.12 to 10.8)	0.783
CHO provided is sub-optimal (yes vs. no)	0.73 (−5.79 to 7.26)	0.826
Study quality (Rosendal score %)	−0.19 (−0.49 to 0.11)	0.211

*PRO* protein, *CHO* carbohydrate, *AA* amino-acids, *kJ* kilojoule

## Discussion

The present systematic review and meta-analysis quantified the effects of consuming CHO (in isolation) and CHO+PRO on the rate of muscle glycogen re-synthesis during short-term post-exercise recovery. Overall, a beneficial effect of ingesting CHO (compared to water or non-nutritive placebo treatment) was observed on the rate of muscle glycogen re-synthesis. However, co-ingestion of CHO with PRO conferred no additional benefit compared to CHO ingested alone. Furthermore, the interval of CHO administration was found to be an influential factor on the rate of muscle glycogen re-synthesis.

### Effect of CHO on Short-Term Muscle Glycogen Re-synthesis

The current meta-analysis suggests that muscle glycogen re-synthesis rate is enhanced during short-term post-exercise recovery when CHO is consumed compared to control (water or non-nutritive placebo treatment). Except for one trial [45], all individual effect estimates indicated a beneficial effect of CHO. However, the magnitude of these effects was heterogeneous (*I*^*2*^ = 66.8).

Meta-regression analysis identified a significant, positive relationship between the magnitude of CHO-induced improvement in muscle glycogen re-synthesis rate and the interval of CHO administration, which explains some of this heterogeneity (*R*^*2*^ = 0.44). Indeed, the regression model for this relationship predicts a ~ 11 mmol·kg dm^−1^ h^−1^ increase in the rate of muscle glycogen re-synthesis when trials administer CHO at an interval of ≤ hourly compared to those administering CHO > hourly. It is worth noting that the amount of CHO consumed is not controlled in this comparison. More frequent CHO administration may enhance muscle glycogen re-synthesis rate by prolonging the elevation of plasma glucose and insulin concentrations [7]. However, to the authors’ knowledge, there are currently no studies (employing a within-subject study design) that have directly assessed the effects of providing equal amounts of CHO at varied frequencies during a fixed period of post-exercise recovery. Nonetheless, the results of this meta-analysis suggest that frequent consumption of CHO (i.e. at least hourly) should be a priority for athletes attempting to optimise short-term muscle glycogen replenishment.

No correlation was observed between the dose of CHO (both relative and total) consumed during post-exercise recovery and rate of muscle glycogen re-synthesis (Table 2). Consequently, we were unable to determine the dose of CHO required to optimise the rate of muscle glycogen re-synthesis. The lack of correlation may be due to the limited number of trials (*n* = 10) available for inclusion in the analysis. As a result, we could not perform multiple meta-regression (due to the limited number of trials) and control for the interval of CHO administration; therefore, this may have prevented the detection of a relationship between CHO dose and muscle glycogen re-synthesis rate. Furthermore, the limited number of trials may have prevented the detection of a relationship between muscle glycogen concentration immediately post-exercise and the rate of muscle glycogen re-synthesis (Table 2). This exploration was of interest because it has previously been hypothesised to have a positive influence (i.e. rate of replenishment is enhanced when post-exercise muscle glycogen concentration is ≤ 150 mmol·kg dm^−1^) on the rate of muscle glycogen re-synthesis, via mechanisms that trigger the insulin-independent phase of muscle glycogen re-synthesis [4, 7].

### Effect of CHO+PRO on Short-Term Muscle Glycogen Re-synthesis

The current meta-analysis suggests that co-ingestion of PRO with CHO during short-term post-exercise recovery provides no additional benefit to (nor does it impair) the rate of muscle glycogen re-synthesis compared to consuming CHO alone. This finding was preserved when contextual factors were explored using meta-regression analysis (Table 4). It is also consistent with results from previous meta-analyses indicating that co-ingestion of PRO with CHO during short-term recovery does not improve short-term muscle glycogen re-synthesis [67] or subsequent exercise performance [68].

Of the 19 trials included in the analysis, only two [29, 58] indicated a significant positive effect favouring CHO+PRO on the rate of muscle glycogen re-synthesis. This result may be due to the co-ingestion of PRO in the context of sub-optimal CHO intake (i.e. ≤ 0.8 g·kg BM^−1^ h^−1^), which has been previously reported as being beneficial [30]. It was suspected this result was due to a large insulinemic response by PRO in combination with CHO, despite inadequate ingestion of the latter. Some research reports a greater insulinemic response when PRO (specifically, containing the AA leucine and phenylalanine) is co-ingested with CHO [4, 7, 30, 69], which has made this an area of interest. This strategy may allow a total reduction in the amount of nutrition needed to stimulate an equivalent insulin response, thus, potentially permitting lower caloric intake while maintaining adequate glycogen re-synthesis. This may be an effective strategy in athletes who are trying to reduce energy consumption (e.g. to make a specific weight division), but need rapid glycogen recovery to maintain subsequent training performance, as well as promote muscle growth and development. However, this strategy is not supported in other trials [27, 53]. The difference amongst trials may be attributed to methodological factors, such as the timing and type of PRO provided (e.g. insulinemic- vs. non-insulinemic-stimulating AA), the mode of exercise performed (e.g. cycling vs. running), and the interval in which muscle tissue was collected between trials (i.e. the length of recovery).

Only one trial [56] showed a significant effect favouring CHO over CHO+PRO on the rate of muscle glycogen re-synthesis. In this trial [56], a mixture of AA were provided, although only in a relatively small dose (0.09 g·kg BM^−1^ h^−1^). The authors hypothesised that the lower rate of muscle glycogen re-synthesis observed in the PRO trial may be due to AA triggering protein synthesis, resulting in glucose being oxidised to support the energy requirement for this process in place of glycogen storage [56]. Nonetheless, while the overall effect of our analysis suggests that co-ingesting PRO with CHO does not provide any benefit beyond that of CHO alone to muscle glycogen restoration (even when CHO intake is suboptimal), it is important to recognise that PRO remains critical for many physiological recovery processes (e.g. muscle repair). As such, a combined CHO+PRO approach is likely to remain an important consideration for nutrition recovery strategies more broadly.

A recent meta-analysis [67] similar to the present study reported a significant main effect (favouring CHO+PRO over CHO) on muscle glycogen re-synthesis rate when the energy intake was not matched between treatments (non-isocaloric). This finding contrasts the results of the present study (Table 4). The discrepancy between findings may be due to a number of factors. Firstly, different effect estimates were used between studies; we reported the mean difference for ease of interpretation [70], whereas Margolis et al. [67] reported Hedges’ *g*. Secondly, studies employing ^13^C-MRS techniques to determine muscle glycogen concentration were included in the previous study, while our results are based on studies using muscle tissue samples for glycogen analysis (as a means of reducing methodological heterogeneity). Thirdly, the previous meta-analysis included studies that provided CHO+PRO in combination with fat, and in some circumstances, the fat content was not matched between treatments [26, 47, 51, 71, 72]. Finally, one study [56] was omitted from the previous meta-analysis without clear explanation and a number of trials [27, 47, 54] that were part of a parallel design were also excluded; in contrast, we included trials from a single study that provided PRO from different sources. As a result, direct comparison of findings between the two meta-analyses is difficult and each should be interpreted on their individual merits.

### Limitations

This review does contain several limitations. Firstly, only studies with accessible full-text articles written in English were included. Secondly, the relatively limited number of trials included in the present meta-analysis prevented a comprehensive exploration of other factors (e.g. CHO type/combinations, dose of CHO/PRO, training status, muscle typology) that can potentially influence the rate of muscle glycogen re-synthesis. The low number of female participants included in original investigations (9.3 and 4.4 % for CHO vs. control and CHO+PRO vs. CHO, respectively) also precluded the exploration of sex as an influential factor on the rate of muscle glycogen re-synthesis. Thus, despite the plethora of research investigating the effect of CHO intake on muscle glycogen re-synthesis, opportunities for further research remain. Studies exploring the influence of sex, muscle fibre typology, training status, CHO type/combination, and the dose of CHO/PRO, on the short-term muscle glycogen re-synthesis, are warranted to enhance our understanding of CHO and CHO+PRO provision during short-term recovery.

## Conclusion

Results of the present review suggest that individuals with limited opportunity for nutritional recovery between consecutive bouts of exercise (e.g. ≤ 8 h) should prioritise CHO ingestion to enhance the rate of muscle glycogen re-synthesis. Co-ingesting PRO with CHO does not appear to enhance the rate of muscle glycogen re-synthesis, nor is it detrimental. The interval of CHO administration appears to be an important factor that may influence the magnitude of effect CHO has on the rate of muscle glycogen re-synthesis. Hence, athletes should be encouraged to consume CHO at least hourly (or more frequently) over the longest period of short-term recovery (≤ 8 h) feasible.

## Supplementary Information

**Additional file 1: Supplementary Table S1.**

**Additional file 2: Supplementary Table S2.**

**Additional file 3: Supplementary Table S3.**

## Data Availability

Not applicable

## Abbreviations

CHO: Carbohydrate
PRO: Protein
BM: Body mass
SD: Standard deviation
AA: Amino acids
dm: Dry mass
MG: Muscle glycogen concentration
*R*: Correlation coefficient

## References

[1] Murray B, Rosenbloom C (2018). Fundamentals of glycogen metabolism for coaches and athletes. Nutr Rev.

[2] Casey A, Mann R, Banister K, Fox J, Morris PG, Macdonald IA (2000). Effect of carbohydrate ingestion on glycogen resynthesis in human liver and skeletal muscle, measured by 13C MRS. Am J Phys.

[3] Johnson NA, Stannard SR, Thompson MW (2004). Muscle triglyceride and glycogen in endurance exercise implications for performance. Sports Med.

[4] Alghannam AF, Gonzalez JT, Betts JA. Restoration of muscle glycogen and functional capacity: role of post-exercise carbohydrate and protein co-ingestion. Nutrients. 2018;10:E253–80. 10.3390/nu10020253 PMC5852829 29473893

[5] Burke LM, van Loon LJC, Hawley JA (2017). Postexercise muscle glycogen resynthesis in humans. J Appl Physiol.

[6] Ivy JL (2004). Regulation of muscle glycogen repletion, muscle protein synthesis and repair following exercise. J Sports Sci Med.

[7] Jentjens R, Jeukendrup AE. Determinants of post-exercise glycogen synthesis during short-term recovery. Sports Med. 2003;33(2):117–. 10.2165/00007256-200333020-00004 12617691

[8] Ivy JL, Lee MC, Brozinick JT, Reed MJ (1988). Muscle glycogen storage after different amounts of carbohydrate ingestion. J Appl Physiol.

[9] Trommelen J, Beelen M, Pinckaers PJM, Senden JM, Cermak NM, van Loon LJC (2016). Fructose coingestion does not accelerate postexercise muscle glycogen repletion. Med Sci Sports Exerc.

[10] Blom PCS, Hostmark AT, Vaage O, Kardel KR, Maehlum S (1987). Effect of different postexercise sugar diets on the rate of muscle glycogen-synthesis. Med Sci Sports Exerc.

[11] Aulin KP, Soderlund K, Hultman E (2000). Muscle glycogen resynthesis rate in humans after supplementation of drinks containing carbohydrates with low and high molecular masses. Eur J Appl Physiol.

[12] Parkin JAM, Carey MF, Martin IK, Stojanovska L, Febbraio MA (1997). Muscle glycogen storage following prolonged exercise: effect of timing of ingestion of high glycemic index food. Med Sci Sports Exerc.

[13] Ivy JL, Katz AL, Cutler CL, Sherman WM, Coyle EF (1988). Muscle glycogen-synthesis after exercise - effect of time of carbohydrate ingestion. J Appl Physiol.

[14] Keizer HA, Kuipers H, van Kranenburg G, Geurten P (1987). Influence of liquid and solid meals on muscle glycogen resynthesis, plasma fuel hormone response, and maximal physical working capacity. Int J Sports Med.

[15] Reed MJ, Brozinick JT, Lee MC, Ivy JL (1989). Muscle glycogen storage postexercise: Effect of mode of carbohydrate administration. J Appl Physiol.

[16] Battram DS, Shearer J, Robinson D, Graham TE (2004). Caffeine ingestion does not impede the resynthesis of proglycogen and macroglycogen after prolonged exercise and carbohydrate supplementation in humans. J Appl Physiol.

[17] Burke LM, Collier GR, Broad EM, Davis PG, Martin DT, Sanigorski AJ (2003). Effect of alcohol intake on muscle glycogen storage after prolonged exercise. J Appl Physiol.

[18] Cheng IS, Huang SW, Lu HC, Wu CL, Chu YC, Lee SD (2012). Oral hydroxycitrate supplementation enhances glycogen synthesis in exercised human skeletal muscle. Br J Nutr.

[19] Impey SG, Hammond KM, Naughton R, Langan-Evans C, Shepherd SO, Sharples AP (2018). Whey protein augments leucinemia and postexercise p70S6K1 activity compared with a hydrolyzed collagen blend when in recovery from training with low carbohydrate availability. Int J Sport Nutr Exerc Metab.

[20] Pedersen DJ, Lessard SJ, Coffey VG, Churchley EG, Wootton AM, Ng T (2008). High rates of muscle glycogen resynthesis after exhaustive exercise when carbohydrate is coingested with caffeine. J Appl Physiol.

[21] Ruby BC, Gaskill SE, Slivka D, Harger SG (2005). The addition of fenugreek extract (Trigonella foenum-graecum) to glucose feeding increases muscle glycogen resynthesis after exercise. Amino Acids.

[22] Slivka D, Cuddy J, Hailes W, Harger S, Ruby B (2008). Glycogen resynthesis and exercise performance with the addition of fenugreek extract (4-hydroxyisoleucine) to post-exercise carbohydrate feeding. Amino Acids.

[23] Tsai TW, Chang CC, Liao SF, Liao YH, Hou CW, Tsao JP (2017). Effect of green tea extract supplementation on glycogen replenishment in exercised human skeletal muscle. Br J Nutr.

[24] Tsao JP, Liao SF, Korivi M, Hou CW, Kuo CH, Wang HF (2015). Oral conjugated linoleic acid supplementation enhanced glycogen resynthesis in exercised human skeletal muscle. J Sports Sci.

[25] Vandoorne T, De Smet S, Ramaekers M, Van Thienen R, De Bock K, Clarke K (2017). Intake of a ketone ester drink during recovery from exercise promotes mTORC1 signaling but not glycogen resynthesis in human muscle. Front Physiol.

[26] Tarnopolsky MA, Bosman M, MacDonald JR, Vandeputte D, Martin J, Roy BD (1997). Postexercise protein-carbohydrate and carbohydrate supplements increase muscle glycogen in men and women. J Appl Physiol.

[27] van Hall G, Saris WHM, van de Schoor PAI, Wagenmakers AJM (2000). The effect of free glutamine and peptide ingestion on the rate of muscle glycogen resynthesis in man. Int J Sports Med.

[28] van Hall G, Shirreffs SM, Calbet JAL (2000). Muscle glycogen resynthesis during recovery from cycle exercise: no effect of additional protein ingestion. J Appl Physiol.

[29] van Loon LJC, Saris WHM, Kruijshoop M, Wagenmakers AJM (2000). Maximizing postexercise muscle glycogen synthesis: carbohydrate supplementation and the application of amino acid or protein hydrolysate mixtures. Am J Clin Nutr.

[30] Betts J, Williams C (2010). Short-term recovery from prolonged exercise. Sports Med.

[31] Potgieter S (2013). Sport nutrition- A review of the latest guidelines for exercise and sport nutrition from the American College of Sport Nutrition, the International Olympic Committee and the International Society for Sports Nutrition. S Afr J Clin Nutr.

[32] Thomas DT, Erdman KA, Burke LM (2016). American College of Sports Medicine Joint Position Statement. Nutrition and Athletic Performance. Med Sci Sports Exerc.

[33] Hickner RC, Fisher JS, Hansen PA, Racette SB, Mier CM, Turner MJ (1997). Muscle glycogen accumulation after endurance exercise in trained and untrained individuals. J Appl Physiol.

[34] Doyle JA, Sherman WM, Strauss RL (1993). Effects of eccentric and concentric exercise on muscle glycogen replenishment. J Appl Physiol.

[35] Casey A, Short AH, Hultman E, Greenhaff PL (1995). Glycogen resynthesis in human muscle-fiber types following exercise-induced glycogen depletion. J Physiol-London.

[36] Piehl K (1974). Time course for refilling of glycogen stores in human muscle fibres following exercise-induced glycogen depletion. Acta Physiol Scand.

[37] Piehl K. Glycogen storage and depletion in human skeletal muscle fibres. Acta Physiol Scand. 1974;90(402 sup):1–32. 4134591

[38] Vollestad NK, Blom PCS, Gronnerod O (1989). Resynthesis of glycogen in different muscle fibre types after prolonged exhaustive exercise in man. Acta Physiol Scand.

[39] Bonen A, Ness GW, Belcastro AN, Kirby RL (1985). Mild exercise impedes glycogen repletion in muscle. J Appl Physiol.

[40] Zachwieja JJ, Costill DL, Pascoe DD, Robergs RA, Fink WJ (1991). Influence of muscle glycogen depletion on the rate of resynthesis. Med Sci Sports Exerc.

[41] Moher D, Shamseer L, Clarke M, Ghersi D, Liberati A, Petticrew M (2015). Preferred reporting items for systematic review and meta-analysis protocols (PRISMA-P) 2015 statement. Syst Rev.

[42] Cheng AJ, Chaillou T, Kamandulis S, Subocius A, Westerblad H, Brazaitis M (2020). Carbohydrates do not accelerate force recovery after glycogen-depleting followed by high-intensity exercise in humans. Scand J Med Sci Sports.

[43] Greene J, Louis J, Korostynska O, Mason A (2017). State-of-the-art methods for skeletal muscle glycogen analysis in athletes-the need for novel non-invasive techniques. Biosensors (Basel).

[44] Areta JL, Hopkins WG (2018). Skeletal muscle glycogen content at rest and during endurance exercise in humans: a meta-analysis. Sports Med.

[45] Haub MD, Potteiger JA, Jacobsen DJ, Nau KL, Magee LA, Comeau MJ (1999). Glycogen replenishment and repeated maximal effort exercise: effect of liquid carbohydrate. Int J Sport Nutr.

[46] Mathai AS, Bonen A, Benton CR, Robinson DL, Graham TE (2008). Rapid exercise-induced changes in PGC-1α mRNA and protein in human skeletal muscle. J Appl Physiol.

[47] Carrithers JA, Williamson DL, Gallagher PM, Godard MP, Schulze KE, Trappe SW (2000). Effects of postexercise carbohydrate-protein feedings on muscle glycogen restoration. J Appl Physiol.

[48] Chandler J, Cumpston M, Li T, Page MJ, Welch VA. Cochrane handbook for systematic reviews of interventions. Wiley Online Library; 2019.

[49] Pascoe DD, Costill DL, Fink WJ, Robergs RA, Zachwieja JJ (1993). Glycogen resynthesis in skeletal muscle following resistive exercise. Med Sci Sports Exerc.

[50] Wilson RJ, Gusba JE, Robinson DL, Graham TE (2007). Glycogenin protein and mRNA expression in response to changing glycogen concentration in exercise and recovery. Am J Physiol Endocrinol Metab.

[51] Roy BD, Tarnopolsky MA (1998). Influence of differing macronutrient intakes on muscle glycogen resynthesis after resistance exercise. J Appl Physiol.

[52] Alghannam AF, Jedrzejewski D, Bilzon J, Thompson D, Tsintzas K, Betts JA (2016). Influence of post-exercise carbohydrate-protein ingestion on muscle glycogen metabolism in recovery and subsequent running exercise. Int J Sport Nutr Exerc Metab.

[53] Betts JA, Williams C, Boobis L, Tsintzas K (2008). Increased carbohydrate oxidation after ingesting carbohydrate with added protein. Med Sci Sports Exerc.

[54] Cogan KE, Evans M, Iuliano E, Melvin A, Susta D, Neff K (2018). Co-ingestion of protein or a protein hydrolysate with carbohydrate enhances anabolic signaling, but not glycogen resynthesis, following recovery from prolonged aerobic exercise in trained cyclists. Eur J Appl Physiol.

[55] Howarth KR, Moreau NA, Phillips SM, Gibala MJ (2009). Coingestion of protein with carbohydrate during recovery from endurance exercise stimulates skeletal muscle protein synthesis in humans. J Appl Physiol.

[56] Wang B, Ding ZP, Wang WY, Hwang JY, Liao YH, Ivy JL (2015). The effect of an amino acid beverage on glucose response and glycogen replenishment after strenuous exercise. Eur J Appl Physiol.

[57] Yaspelkis BB, Ivy JL (1999). The effect of a carbohydrate-arginine supplement on postexercise carbohydrate metabolism. Int J Sport Nutr.

[58] Zawadzki KM, Yaspelkis BB, Ivy JL (1992). Carbohydrate-protein complex increases the rate of muscle glycogen storage after exercise. J Appl Physiol.

[59] Beelen M, Van Kranenburg J, Senden JM, Kuipers H, Van Loon LJC (2012). Impact of caffeine and protein on postexercise muscle glycogen synthesis. Med Sci Sports Exerc.

[60] Jentjens R, Van Loon LJC, Mann CH, Wagenmakers AJM, Jeukendrup AE (2001). Addition of protein and amino acids to carbohydrates does not enhance postexercise muscle glycogen synthesis. J Appl Physiol.

[61] Jadad AR, Moore RA, Carroll D, Jenkinson C, Reynolds DJ, Gavaghan DJ (1996). Assessing the quality of reports of randomized clinical trials- is blinding necessary?. Control Clin Trials.

[62] Higgins JP, Green S (2011). Cochrane Handbook for Systematic Reviews of Interventions. Collaboration TC, editor.

[63] Higgins JPT, Thompson SG, Deeks JJ, Altman DG (2003). Measuring inconsistency in meta-analyses. Br Med J.

[64] Gusba JE, Wilson RJ, Robinson DL, Graham TE (2008). Interleukin-6 and its mRNA responses in exercise and recovery: relationship to muscle glycogen. Scand J Med Sci Sports.

[65] Adamo KB, Tarnopolsky MA, Graham TE (1998). Dietary carbohydrate and postexercise synthesis of proglycogen and macroglycogen in human skeletal muscle. Am J Physiol Endocrinol Metab.

[66] Lunn WR, Pasiakos SM, Colletto MR, Karfonta KE, Carbone JW, Anderson JM (2012). Chocolate milk and endurance exercise recovery: protein balance, glycogen, and performance. Med Sci Sports Exerc.

[67] Margolis LM, Allen JT, Hatch-McChesney A, Pasiakos SM. Coingestion of Carbohydrate and Protein on Muscle Glycogen Synthesis after Exercise: A Meta-analysis. Med Sci Sports Exerc. 2020. 10.1249/mss.0000000000002476. 10.1249/MSS.0000000000002476 PMC7803445 32826640

[68] McCartney D, Desbrow B, Irwin C (2018). Post-exercise ingestion of carbohydrate, protein and water: a systematic review and meta-analysis for effects on subsequent athletic performance. Sports Med.

[69] van Loon LJC, Kruijshoop M, Verhagen H, Saris WHM, Wagenmakers AJM (2000). Ingestion of protein hydrolysate and amino acid-carbohydrate mixtures increases postexercise plasma insulin responses in men. J Nutr.

[70] Takeshima N, Sozu T, Tajika A, Ogawa Y, Hayasaka Y, Furukawa TA (2014). Which is more generalizable, powerful and interpretable in meta-analyses, mean difference or standardized mean difference?. BMC Med Res Methodol.

[71] Williams MB, Raven PB, Fogt DL, Ivy JL (2003). Effects of recovery beverages on glycogen restoration and endurance exercise performance. J Strength Cond Res.

[72] Kammer L, Ding ZP, Wang B, Hara D, Liao YH, Ivy JL (2009). Cereal and nonfat milk support muscle recovery following exercise. J Int Soc Sports Nutr.

