# Supplementary material for: The Effect of Consuming Carbohydrate With and Without Protein on the Rate of Muscle Glycogen Re-synthesis During Short-Term Post-exercise Recovery: a Systematic Review and Meta-analysis
Source: Sports Med Open. 2021 Jan 28;7:9. doi: 10.1186/s40798-020-00297-0 (PMC7843684; doi:10.1186/s40798-020-00297-0)
Supplement: Supplementary file 1 — Additional file 1: Supplementary Table S1. [file 40798_2020_297_MOESM1_ESM.docx]

**Table S1.** Study quality assessment

| **Citation** | A clear description of the inclusion and exclusion criteria was provided | The trials were randomized | Treatment order was counterbalanced | The method used to generate the random allocation sequence was described | Sample size was justified | Attempts were made to control and/or monitor pre-trial conditions | Design incorporated measures of important baseline variables | Subjects were blinded | Investigators were blinded | Methods and successfulness of blinding were described | Details were provided regarding the inability of a subject to complete study requirements | Statistical methods described | Primary outcome measurement and variability reported | Results of statistical comparisons reported | Methods used to assess adverse effects described | Reproducibility of the primary outcome measure(s) was reported | A familiarization of the performance test was conducted | **Total Score (%)** |
| --- | --- | --- | --- | --- | --- | --- | --- | --- | --- | --- | --- | --- | --- | --- | --- | --- | --- | --- |
| **Studies Investigating CHO vs. Control** | | | | | | | | | | | | | | | | | | |
| Cheng et al. 2020 | 1 | 1 | 0 | 0 | 0 | 1 | 1 | NA | NA | NA | 1 | 1 | 1 | 1 | 0 | 0 | 1 | 69 |
| Haub et al. 1999 | 1 | 1 | 1 | 0 | 0 | 1 | 1 | NA | NA | NA | NA | 1 | 1 | 1 | 0 | 0 | 1 | 69 |
| Ivy et al. 1988 | 0 | 1 | 0 | 0 | 0 | 1 | 1 | NA | NA | NA | NA | 1 | 1 | 1 | 0 | 0 | 1 | 54 |
| Mathai et al. 2008 | 0 | 1 | 0 | 0 | 0 | 1 | 1 | NA | NA | NA | 1 | 1 | 1 | 1 | 0 | 0 | 1 | 57 |
| Pascoe et al. 1993 | 1 | 1 | 0 | 0 | 0 | 1 | 1 | NA | NA | NA | NA | 1 | 1 | 1 | 0 | 0 | 1 | 62 |
| Roy et al. 1998 | 1 | 1 | 0 | 0 | 0 | 1 | 1 | NA | NA | NA | NA | 1 | 1 | 1 | 0 | 0 | 0 | 54 |
| van Hall et al. 2000 | 0 | 1 | 0 | 0 | 0 | 1 | 1 | NA | NA | NA | 1 | 1 | 1 | 1 | 0 | 0 | 0 | 50 |
| Tarnopolsky et al.1997 | 1 | 1 | 0 | 0 | 0 | 1 | 1 | NA | NA | NA | NA | 1 | 1 | 1 | 0 | 0 | 1 | 62 |
| Wilson et al. 2007 | 0 | 1 | 0 | 0 | 0 | 1 | 1 | NA | NA | NA | NA | 1 | 1 | 1 | 0 | 0 | 1 | 54 |
| **Studies Investigating CHO+PRO vs. CHO** | | | | | | | | | | | | | | | | | | |
| Alghannam et al. 2016 | 0 | 1 | 1 | 0 | 1 | 1 | 1 | NA | NA | NA | NA | 1 | 1 | 1 | 1 | 0 | 1 | 77 |
| Beelen et al. 2011 | 1 | 1 | 0 | 0 | 0 | 1 | 1 | NA | NA | NA | NA | 1 | 1 | 1 | 1 | 0 | 0 | 62 |
| Betts et al. 2008 | 1 | 1 | 0 | 0 | 1 | 1 | 1 | NA | NA | NA | NA | 1 | 1 | 1 | 1 | 0 | 1 | 77 |
| Carrithers et al. 2000 | 1 | 1 | 0 | 0 | 0 | 1 | 1 | NA | NA | NA | 1 | 1 | 1 | 1 | 0 | 0 | 1 | 64 |
| Cogan et al. 2018 | 1 | 1 | 0 | 0 | 0 | 1 | 1 | NA | NA | NA | NA | 1 | 1 | 1 | 0 | 0 | 0 | 54 |
| Howarth et al. 2009 | 1 | 1 | 0 | 0 | 0 | 1 | 1 | NA | NA | NA | NA | 1 | 1 | 1 | 1 | 0 | 0 | 62 |
| Jentjens et al. 2001 | 1 | 1 | 0 | 0 | 0 | 1 | 1 | NA | NA | NA | 1 | 1 | 1 | 1 | 1 | 0 | 0 | 64 |
| van Hall et al. 2000 | 0 | 1 | 0 | 0 | 0 | 1 | 1 | NA | NA | NA | 1 | 1 | 1 | 1 | 0 | 0 | 0 | 50 |
| van Hall et al. 2000 | 1 | 1 | 0 | 0 | 0 | 1 | 1 | NA | NA | NA | NA | 1 | 1 | 1 | 0 | 0 | 0 | 54 |
| van Loon et al. 1999 | 1 | 1 | 0 | 0 | 0 | 1 | 1 | NA | NA | NA | NA | 1 | 1 | 1 | 0 | 0 | 0 | 54 |
| Wang et al. 2015 | 1 | 1 | 1 | 0 | 0 | 1 | 1 | NA | NA | NA | NA | 1 | 1 | 1 | 1 | 0 | 1 | 77 |
| Yaspelkis III et al. 1999 | 0 | 1 | 0 | 0 | 0 | 1 | 1 | NA | NA | NA | NA | 1 | 1 | 1 | 0 | 0 | 1 | 54 |
| Zawadzki et al. 1992 | 1 | 1 | 0 | 0 | 0 | 1 | 1 | NA | NA | NA | NA | 1 | 1 | 1 | 0 | 0 | 1 | 62 |
