# Supplementary material for: The Effect of Consuming Carbohydrate With and Without Protein on the Rate of Muscle Glycogen Re-synthesis During Short-Term Post-exercise Recovery: a Systematic Review and Meta-analysis
Source: Sports Med Open. 2021 Jan 28;7:9. doi: 10.1186/s40798-020-00297-0 (PMC7843684; doi:10.1186/s40798-020-00297-0)
Supplement: Supplementary file 2 — Additional file 2: Supplementary Table S2. [file 40798_2020_297_MOESM2_ESM.docx]

**Table S2.** Sensitivity analysis of alternative levels of correlation coefficient (*R*) on overall CHO vs. Control meta-analysis results

| ***R*** | **MG_Δ_ re-synthesis rate (mmol·kg dm^−1^·h^−1^)**  **(95% CI)** | ***p* value** | ***I*^2^ index** |
| --- | --- | --- | --- |
| **Actual** |  |  |  |
| 0.28 | 23.5 (19.0, 27.9) | <0.0001 | 66.8 |
| **Alternative** |  |  |  |
| 0.90 | 26.6 (22.1, 31.0) | <0.0001 | 92.2 |
| 0.60 | 24.5 (20.1, 28.4) | <0.0001 | 79.1 |
