# Supplementary material for: The Effect of Consuming Carbohydrate With and Without Protein on the Rate of Muscle Glycogen Re-synthesis During Short-Term Post-exercise Recovery: a Systematic Review and Meta-analysis
Source: Sports Med Open. 2021 Jan 28;7:9. doi: 10.1186/s40798-020-00297-0 (PMC7843684; doi:10.1186/s40798-020-00297-0)
Supplement: Supplementary file 3 — Additional file 3: Supplementary Table S3. [file 40798_2020_297_MOESM3_ESM.docx]

**Table S3.** Sensitivity analysis of alternative levels of correlation coefficient (*R*) on overall CHO+PRO vs. CHO meta-analysis results

| ***R*** | **MG_Δ_ re-synthesis rate (mmol·kg dm^−1^·h^−1^)**  **(95% CI)** | ***p* value** | ***I*^2^ index** |
| --- | --- | --- | --- |
| **Actual** |  |  |  |
| 0.28 | 0.4 (-2.7, 3.4) | 0.805 | 56.4 |
| **Alternative** |  |  |  |
| 0.90 | 0.7 (-2.6, 4.0) | 0.691 | 84.7 |
| 0.60 | 0.6 (-2.5, 3.8) | 0.699 | 69.0 |
